# Nasopharyngeal carcinoma: molecular biomarker discovery and progress

**DOI:** 10.1186/1476-4598-6-1

**Published:** 2007-01-02

**Authors:** William Chi-shing Cho

**Affiliations:** 1 Department of Clinical Oncology, Queen Elizabeth Hospital, Room 1305, 13/F, Block R, 30 Gascoigne Road, Kowloon, Hong Kong SAR, PR China

## Abstract

Nasopharyngeal carcinoma (NPC) is a rare malignancy in most part of the world and it is one of the most confusing, commonly misdiagnosed and poorly understood diseases. The cancer is an Epstein-Barr virus-associated malignancy with a remarkable racial and geographical distribution. It is highly prevalent in southern Asia where the disease occurs at a prevalence about a 100-fold higher compared with other populations not at risk. The etiology of NPC is thought to be associated with a complex interaction of genetic, viral, environmental and dietary factors. Thanks to the advancements in genomics, proteomics and bioinformatics in recent decades, more understanding of the disease etiology, carcinogenesis and progression has been gained. Research into these components may unravel the pathways in NPC development and potentially decipher the molecular characteristics of the malignancy. In the era of molecular medicine, specific treatment to the potential target using technologies such as immunotherapy and RNAi becomes formulating from bench to bedside application and thus makes molecular biomarker discovery more meaningful for NPC management. In this article, the latest molecular biomarker discovery and progress in NPC is reviewed with respect to the diagnosis, monitoring, treatment and prognostication of the disease.

## Background

Nasopharyngeal carcinoma (NPC) is a disease in which malignant cells form in the tissues of the nasopharynx. As one of the most common cancers among Chinese or Asian ancestry, it poses one of the serious health problems in southern China where an annual incidence of more than 20 cases per 100,000 is reported. Men are twice as likely to develop NPC as women. The rate of incidence generally increases from ages 20 to around 50. Signs and symptoms at presentation include painless, enlarged cervical lymph nodes, nasal obstruction, epistaxis, diminished hearing, tinnitus, recurrent otitis media, cranial nerve dysfunction, sore throat and headache. According to the tumour-node-metastasis staging system promulgated by the American Joint Committee on Cancer, patients are designated into stages 0, I, IIA, IIB, III, IVA, IVB and IVC.

## Molecular biomarkers in etiology

Factors thought to predispose to NPC include ethnic background, Epstein-Barr virus (EBV) exposure and consumption of food with volatile nitrosamines [1]. It was found that the polymorphism of a nitrosamine metabolizing gene, *CYP2A6*, might play a crucial role in NPC susceptibility and it might be used as a risk marker for NPC [2].

In the most prevalent area, a lot of genetic polymorphism of *CYP2F1 *gene is found in Guangdong population of China. When the genetic polymorphism of *CYP2F1 *was investigated, the cooperated operations with multiple genetic polymorphisms of one or more genes were found to be potential critical factors contributing to the development and progression of NPC [3]. On the other hand, the *XRCC1 *gene is important in DNA base excision repair. It was hypothesized that two common single nucleotide polymorphisms of *XRCC1 *(codons 194 Arg → Trp and 399 Arg → Gln) were related to the risk of NPC and interacted with tobacco smoking. The *XRCC1 *Trp194Trp variant genotype was found to be associated with a reduced risk of developing NPC in Guangdong population, particularly in males and smokers [4]. On the other hand, *Cyclin D1 *(a key regulator of the cell cycle) and its altered activity associated with the development of cancer has been studied in a low-risk country. The proportion of NPC cases attributable to the GG *Cyclin D1 *genotype was 15% in Portuguese patients with NPC. This result might be important in the definition of a biologic predictive profile for the development of NPC within the Portugal population [5].

## Molecular biomarkers of carcinogenesis

NPC is a complex disease caused by an interaction of EBV chronic infection, environment and host genes, in a multi-step process of carcinogenesis. Genomic instability can be an early event marker in carcinogenesis of NPC. Aggravation of genomic alterations is also a poor prognosis for cancer recovery [6]. For understanding the putative order of genetic alteration in NPC carcinogenesis, evolutionary tree models (branching and distance-based tree models) were used to analyze comparative genomic hybridization data of NPC cases. Chromosome 12 gain and consistent loss of 3p for both tree models were important early events in NPC progression. The tree models suggested two subclasses of 3p-derived NPC, one marked by 1q+, 9p- and 13q-, and the other marked by 14q-, 16q-, 9q- and 1p- [7].

Using bioinformatics to analyze the expression and location of UBAP1 protein, it was found that EGFP/UBAP1 was expressed and existed mainly in the nuclear, especially accumulated on the nuclear envelope. The expression difference in NPC cells might be related to the carcinogenesis of NPC [8]. Moreover, a locus on 3p21 was identified to link to NPC in a linkage analysis [9]. *RASSF1A *is a tumour suppressor gene on 3p21.3 frequently inactivated by promoter hypermethylation in NPC. Investigated by high-density oligonucleotide array, the expressions of *activin βE *and *Id2 *in NPC were tightly regulated by *RASSF1A*. *RASSF1A*-induced repression of *Id2 *was mediated by the overexpression of *activin βE*. The results suggested a novel *RASSF1A *pathway in which both *activin βE *and *Id2 *were involved [10].

A new carcinoma-related gene named *KIAA1173 *locating at 3p22.1 was also characterized. It was strongly expressed in normal nasopharyngeal mucosa epithelia, but downregulated in NPC. *KIAA1173 *might be associated with the carcinogenesis of NPC [11]. In a study using microdissection and cDNA microarray, gene expression patterns suggested the dysregulation of the GTP/GDP-bound *Ras *cycle and an abnormal hyperactivity of cell cycle in NPC. Alterations in the Wingless-type (Wnt) pathway suggested that this pathway might be activated in NPC [12]. On the other hand, elevation of plasma osteopontin level was found in patients with undifferentiated NPC. The finding indicated a potential role of osteopontin in the pathogenesis and nodal metastasis of undifferentiated NPC [13].

## Molecular diagnostic biomarkers

Diagnosis is made by biopsy of the nasopharyngeal mass. Fused positron emission tomography/computed tomography is a valuable imaging tool in patients for staging diagnosis of NPC. However, NPC is commonly diagnosed late due to its deep location and vague symptoms [14]. By measuring the nuclear DNA content, DNA diploidy was found to occur earlier in the progression from premalignant to malignant head and neck squamous cell carcinomas (including NPC). This finding was promising to demonstrate methods that were readily applicable for routine diagnostic work [15]. Elevated RNase activity has previously been described in the circulation of cancer patients, and NPC was found to be associated with disturbances in the integrity of cell-free circulating RNA. Measurement of plasma RNA integrity might serve as a useful marker for the diagnosis and monitoring of NPC [16].

It has been reported that the high sensitivity (81%) and specificity (0% false positives) of detecting aberrant methylation of *CDH13 *(encoded a cell adhesion molecule H-cadherin) from nasopharyngeal swabs suggested it could be utilized as a tool for early diagnosis [17]. Systematically identified by sodium dodecyl sulfate polyacrylamide gel electrophoresis combined with matrix-assisted laser desorption/ionization time-of-flight mass spectrometry and further confirmed by Western blot analysis in the NPC cell-lines, fibronectin, Mac-2 binding protein and plasminogen activator inhibitor 1 were found to be potential markers for diagnosis of NPC [18] (Table 1).

## Molecular biomarkers of targeted therapies

High-dose radiotherapy with adjunctive chemotherapy is the primary treatment of NPC [19]. Surgery, when feasible, is usually reserved for nodes that fail to regress after radiotherapy and chemotherapy, or for nodes that recurrent following clinical complete response. Radiotherapy dose and field margins are individually tailored to the location and size of the primary tumour and lymph nodes [20,21]. New types of treatment are being tested in clinical trials, which include biological therapy and intensity-modulated radiation therapy. Advances in immunologic research and combined chemotherapy offer hope for better control of the disease [22].

Using oligonucleotide microarray analysis mapping close to a previously defined 11q22-23 NPC critical region, *THY1 *showed consistent downregulated expression in the tumour segregants. *THY1 *was identified to be a candidate tumour suppressor gene significantly associated with lymph node metastatic NPC [23]. Employing the monochromosome transfer approach, it was shown that chromosome 3p could suppress tumour growth *in vivo*. By quantitative reverse transcription polymerase chain reaction (PCR), a candidate tumour suppressor gene *BLU/ZMYND10 *mapping in the 3p21.3 critical region, was frequently downregulated in NPC cell lines and NPC biopsies [24]. Another tumour suppressor gene *DLC-1*, locating at the human chromosome region 8p22, is frequently deleted in NPC. The mRNA level of *DLC-1 *was found to be downregulated in NPC. To identify the mechanism of *DLC-1 *downregulation, the methylation status of *DLC-1 *was investigated using methylation-specific PCR. Results showed that *DLC-1 *might be a NPC-related tumour suppressor gene affected by aberrant promoter methylation and gene deletion [25].

Stress-responsive gene *GADD45G *was found to be a functional new-age tumour suppressor, with its response to environmental stresses frequently disrupted epigenetically in NPC [26]. Another study suggested that a novel bromodomain gene, *BRD7*, was identified to be associated with NPC. Overexpression of *BRD7 *could inhibit NPC cell growth and arrest cells in cell cycle by transcriptionally regulating some important molecules involved in *ras*/*MEK*/*ERK *and *Rb*/*E2F *pathway, and downregulate the promoter activity of *E2F3*. The nuclear localization of *BRD7 *was critical for the expression of cell cycle related molecules and cell biological function [27]. Using colony formation assay, Cheung *et al *found a suppression of human *MAD2B *conferred hypersensitivity to a range of DNA-damaging agents, especially DNA cross-linkers, such as cisplatin and gamma-irradiation. The result indicated that cancer cells were sensitized to DNA-damaging anticancer drugs through inactivation of *MAD2B *in NPC [28].

Constitutive activation of Wnt signaling and *WIF-1 *silencing was found in NPC cell lines. Utilizing methylation-specific PCR and sequence analysis, frequent hypermethylation of the *WIF-1 *promoter correlated with *WIF-1 *silencing was demonstrated in NPC cell lines. These results indicated that aberrant Wnt signaling was a common event in NPC carcinogenesis linked with *WIF-1 *silencing in cell lines. Strategies targeting these molecules might be potentially promising in treating NPC [29]. The 14-3-3σ gene product, up-regulated by *p53 *in response to DNA damage, is involved in cell-cycle checkpoint control and is a human cancer epithelial marker downregulated in various tumours. It was reported that overexpression of 14-3-3σ in NPC cell lines reduced the tumour volume in nude mice. This finding provided an insight into the role of 14-3-3σ in NPC and suggested that modulating 14-3-3σ activity might be useful in the treatment of NPC [30].

Using immunohistochemical streptomycin-avidin peroxidase staining and terminal deoxynucleotidyl transferase mediate dUTP nick and labeling technique, He and Kong demonstrated that the expression of effector cell protease receptor-1 played a role in increase the apoptosis and decrease the proliferation of cell in NPC. This suggested that effector cell protease receptor-1 might be a potential therapy for NPC [31]. Death-associated protein kinase (DAPK) is a Ca/calmodulin-regulated serine/threonine kinase and a positive mediator of apoptosis. Loss of DAPK expression was shown to be associated with promoter region methylation in NPC. A demethylating agent, 5-Aza-2'-deoxycytidine, might slow the growth of NPC cells *in vitro *and *in vivo *by reactivating the *DAPK *gene silenced by *de novo *methylation [32]. The antiapoptotic gene *bcl-2 *antisense oligodeoxynucleotide, G3139, was found to have proapoptotic effects in C666-1 cell line. Combining with cisplatin, it was curative in C666-1 NPC xenograft tumours *in vivo*. The sequence-dependency of these effects was consistent with an antisense mechanism. The result suggested that *bcl-2 *might represent a biologically relevant target for the development of novel combinatorial therapies for NPC [33].

The discovery of RNA interference (RNAi) gene silencing by double-stranded RNA earned the Nobel Prize in Physiology or Medicine in 2006 and led the treatment of disease to a new horizon. The transient transfected *bcl-xL *siRNA4 could effectively inhibit the growth of the cancer cells and induce their apoptosis. Knockdown of *bcl-xL *expression with RNAi induced NPC cells apoptosis, suggesting that the siRNA technique could provide a new method for anti-NPC gene therapy [34]. Epidermal growth factor receptor silencing by RNAi could reduce the proliferation of NPC cells and induce cell cycle arrest at G1 phase, which shed light on the possible use of RNAi for further investigation of the pathogenesis and gene therapy of NPC [35]. Pathway analyses by microarrays revealed that upregulation of NF-κB2 and survivin played central roles in increasing resistance to apoptosis, as well as changes in integrin and Wnt/β-catenin signaling leading to uncontrolled proliferation. The role of survivin in resisting apoptosis in NPC was confirmed by RNAi, which suggested survivin as a novel therapeutic target for NPC [36].

## Molecular biomarkers for treatment response monitoring

Major factors adversely influencing outcome of treatment include large size of the tumour, advanced tumour stage and the presence of involved cervical lymph nodes [37,38]. The DNA anueploid content in NPC was found to be positively related to the S phase cells by flow cytometric analysis. Patients having a low expression of Ki67 or DNA aneuploid in tumour cells were not sensitive to chemotherapy, liable to metastasis to distant organs and had a poor prognosis. It was suggested that DNA ploidy and Ki67 could be used as an independent and objective marker to evaluate the radiosensitivity and prognosis of NPC [39]. The changes of serum vascular endothelial growth factor (VEGF) before and after radiotherapy in NPC patients were studied. Zhao *et al *reported that patients with high serum VEGF level were found to have a poor prognosis [40]. Endothelin-1 is a potent vasoactive peptide and a hypoxia-inducible angiogenic growth factor associated with the development and spread of solid tumours. Pretreatment plasma big endothelin-1 levels might be useful in predicting posttreatment distant failure in patients with advanced-stage NPC [41].

Ceruloplasmin (CPL) was identified as a potential serum biomarker by mass spectrometric analysis and MASCOT database search. The enhanced expression of CPL in the NPC patients' sera was confirmed by competitive enzyme-linked immunosorbent assay (ELISA). When follow-up two-dimensional electrophoresis and ELISA studies were performed on the NPC patients who responded positively to treatment, the difference in CPL expression was no longer significant [42] (Table 1).

Using proteinchip profiling analysis, two isoforms of serum amyloid A (SAA) protein were identified as useful biomarkers to monitor relapse of NPC. Monitoring the patients longitudinally for SAA level by proteinchip and validated by immunoassay showed a dramatic SAA increase, which correlated with relapse and a drastic fall correlated with response to salvage chemotherapy [43]. Further examination was conducted to found other serum biomarkers that were associated with active disease or chemotherapy response in NPC patients treated by two different drug combinations. Using tandem MS sequencing and immunoaffinity capture assay, two potential biomarkers were identified as a fragment of inter-α-trypsin inhibitor precursor and platelet factor-4. These disease and treatment associated serum biomarkers might serve to diagnose and triage NPC patients for appropriate chemotherapy treatment respectively [44] (Table 1).

## Molecular biomarkers for prognosis and progression of cancer

Small cancers of the nasopharynx are highly curable by radiotherapy with chemotherapy and have shown survival rates of 80% to 90% [45]. Moderately advanced lesions without clinical evidence of spread to cervical lymph nodes are often curable and have shown survival rates of 50% to 70%. Patients with advanced lesions, especially those associated with clinically positive cervical lymph nodes, cranial nerve involvement and bone destruction, are poorly controlled locally by radiotherapy with or without surgery and often develop distant metastases despite local control [46,47]. Although most recurrences occur within five years of diagnosis, relapse can be seen at longer intervals [48].

It has been reported that average expression of *Tiam1 *in NPC tissue was higher than in normal nasopharyngeal tissue. This data suggested that the overexpression of the *Tiam1 *correlated invasion and metastasis of NPC [49]. Interleukin IL-8 receptor A was demonstrated to be overexpressed in tumour cells and correlated significantly with angiogenesis in NPC. The result suggested that the expression of IL-8 receptor A in tumour cells might be an important indicator of poor prognosis in NPC [50]. The positive gradual expression of estrogen and progestogen receptors in NPC was well correlated with distant metastasis. Strong positive expression pointed out bad prognosis and endocrine treatment might reduce and postpone distant metastasis [51]. Adopting immunohistochemistry labeled streptavidin biotin method, overexpression of epidermal growth factor receptor and phosphorylated extracellular signal-regulated kinase was detected in NPC. The abnormally high expression signified poor prognosis in NPC patients [52].

VEGF expression was assessed in NPC and benign adenoid lesions by immunohistochemistry and EBV presence by PCR using primers directed against EBV nuclear antigen EBNA-1. The results pointed towards the potential of the expression pattern of VEGF as a tumour marker for the early diagnosis of metastatic NPC and also showed that presence of EBV was related to up regulation of VEGF [53]. An immunohistochemistry study found that VEGF and its receptors fms-like tyrosine kinase-1 and kinase insert domain containing receptor were widely expressed in NPC tissues. Their expressions were positively related to clinical features and prognosis of NPC patients [54]. The expressions of nm23-H1 and VEGF protein were examined by immunohistochemistry S-P staining in NPC tissues. The low level expression of nm23-H1 protein and the high level expression of VEGF protein might be associated with the development and poor prognosis of NPC [55].

NPC samples expressed high levels of survivin and livin, which might play an important role in the oncogenesis and tumour development. Overexpression of survivin was related with poor prognosis, suggesting that the determination of survivin expression might provide predictive information on NPC patients [56]. It has been reported that high Bmi-1 oncoprotein expression was found to be positively correlated with poor prognosis of NPC patients. This finding suggested that Bmi-1 played an important role in the development and progression of NPC, and that it was a valuable marker for assessing the prognosis of NPC patients [57]. Immunohistochemistry was performed on formalin-fixed paraffin-embedded sections of patients with NPC. Bar-Sela *et al *found that heparanase expression was inversely correlated with survival of NPC patients, clearly indicating that heparanase was a reliable prognostic factor for this malignancy [58].

## Molecular biomarkers of Epstein-Barr virus-associated nasopharyngeal carcinoma

EBV is an oncogenic human gamma-herpesvirus that persistently infects more than 90% of the human population. There are compelling evidences suggesting that EBV is a causal agent of NPC and is most likely to be involved in the multi-step and multi-factorial development of the cancer. EBV encoded genes have been shown to be involved in immune evasion and in the regulation of various cellular signaling cascades. The fact that EBV genome is present in almost all NPC tissues renders it an ideal tumour marker for NPC. Quantitative analyses of EBV antibodies and EBV DNA have been shown to be clinically useful for the early detection, monitoring and prognostication of NPC.

Assessment of immunoglobulin A (IgA) and immunoglobulin G (IgG) antibodies responses to various EBV antigen complexes, usually involving multiple serological assays, is important for the early diagnosis of NPC. EBNA-1, the viral protein uniformly expressed in NPC, represents a prime target for T-cell based immunotherapy [59]. Through combination of two synthetic peptides representing immunodominant epitopes of EBNA-1 and viral capsid antigen VCA-p18, a one-step sandwich ELISA for the specific detection of EBV reactive IgA and IgG antibodies in NPC patients was developed [60]. Comparing the antibody levels to VCA of EBV as potential diagnostic markers of NPC, VCA-IgA had an advantage over VCA-IgG despite the slightly lower sensitivity due to its consistently more distinct fluorescence reaction [61]. In a combination of the surface-enhanced laser desorption/ionization time-of-flight mass spectrometry serum protein profiles with EBNA-1 IgA test, the diagnostic sensitivity and specificity were increased to 99% and 96% respectively [62]. The results of immunoprecipitation suggested a direct interaction between EBNA-5 and p63 protein in NPC, and this binding would increase the stability of p63. It was suggested that p63 might be used as an adjunct diagnostic marker of NPC and contributed a new way to understand the contribution of the EBV in the pathogenesis of NPC [63]. Dynamic detection of serum sialic acid and VCA-IgA might be a valuable technique for diagnosis and monitoring radiotherapy effectiveness in NPC patients. The combined determination of the two indexes could raise the positive rate of patients with NPC [64]. Poorly differentiated squamous cancer was found to be associated with EBV antibodies [65]. High-titer antibodies to VCA and early antigen, especially of high IgA class, or high titers that persist after therapy, were found to be associated with a poorer prognosis [66].

The molecular nature of circulating EBV DNA has been identified as free DNA fragments, and it was not contained inside intact virions. By quantitative size analysis, Chan and Lo demonstrated that more than 80% of these DNA fragments were less than 180 bp in size [67]. In the comparison of EBV DNA levels in plasma and peripheral blood cell in NPC patients, plasma EBV DNA derived from the cancer cells was more sensitive and reliable than peripheral blood cell EBV DNA from circulating mononuclear cells for diagnosis, staging and therapeutic effect evaluation at a molecular level in NPC clinical practice [68]. The detection of plasma EBV DNA could reflect the tumours growth and decline. It was an important and sensitive index in diagnosing the residual and relapse of NPC [69]. Plasma EBV DNA concentration could be used to predict distant metastasis in NPC. The detecting rates of both pre-treatment and post-treatment EBV DNA concentrations in patients with distant metastasis were significantly higher than those with continuous remission and those with local relapse [70]. The plasma EBV DNA load was shown to be proportionately related to the presence of NPC. This finding underscored the prognostic value of cell-free EBV DNA quantification [71].

There was a study showing that consecutive patients with metastatic or recurrent NPC receiving combination chemotherapy were monitored for EBV DNA in their serum. Profile of EBV encoded RNA (EBER-1) DNA showed concordance with clinical course of either continuous remission or later progression. EBER-1 DNA in serum could become a useful adjunctive surrogate marker to monitor chemotherapeutic response in NPC patients with distant metastasis or advanced local recurrence [72]. Differential expression of EBER and several tumour-related genes were found in NPC using tissue microarray analysis. EBV infection, together with overexpression of p53, and loss expressions of p16 and p27 proteins were involved in the multistep process of human nasopharyngeal epithelial carcinogenesis [73].

The EBV oncogene *BARF1 *is expressed in a high proportion of NPC. The structure of the secreted BARF1 glycoprotein expressed in a human cell line was solved by X-ray crystallography. It was most closely related to CD80 or B7-1, a co-stimulatory molecule present on antigen presenting cells, from which *BARF1 *was derived during evolution [74]. Measurement of EBV DNA load combined with *BARF1 *mRNA detection in simple nasopharyngeal brushings allowed non-invasive NPC diagnosis. It reflected carcinoma-specific EBV involvement at the anatomical site of tumour development and reduced the need for invasive biopsies. This procedure might be useful for confirmatory diagnosis in large serological NPC screening program [75].

*In vitro *EBV infection resulted in the activation of STAT3 and NF-κB signal cascades in nasopharyngeal epithelial cells. Increased expression of their downstream targets (*c-Myc*, *bcl-xL*, *IL-6*, *LIF*, *SOCS-1*, *SOCS-3*, *VEGF *and *COX-2*) was also observed. EBV latent infection induced the suppression of *p38-MAPK *activities, but did not activate *PKR *cascade. These findings suggested that EBV latent infection was able to manipulate multiple cellular signal cascades to protect infected cells from immunologic attack and to facilitate cancer development [76]. Measuring the expression of latent EBV genes in NPC and normal nasopharyngeal tissue samples, it was shown that deregulation of key proteins involving in apoptosis (bcl-2 related protein A1 and Fas apoptotic inhibitory molecule), cell cycle checkpoints (AKIP, SCYL1 and NIN) and metastasis (matrix metalloproteinase 1) were closely correlated with the levels of EBV gene expression in NPC [77].

Of the EBV-encoded product, latent membrane protein *LMP-1 *is considered to be an oncogene playing an essential role in cell transformation and metastasis. It is necessary for EBV-induced transformation of B lymphocytes and is able to transform Rat-1 fibroblasts. LMP-1 can activate a wide array of signaling pathways, including phosphatidylinositol 3-kinase-Akt and NF-κB. It was found that the signature amino acid changes of the LMP-1 variants did not hinder or enhance their *in vitro *transforming potentials or affect their signaling properties [78]. Combing the novel strategy of phosphoprotein enrichment with proteomics technology to elucidate the signaling cascade activated by LMP-1, annexin A2, heat shock protein 27, stathmin, annexin I, basic transcription factor 3 and porin were identified to be novel signaling molecules or targets with no previously known function in LMP-1 signal transduction [79] (Table 1). Pilot study of LMP-1 and CD99 expressions in NPC suggested that the LMP-1 induced down-regulation of the CD99 pathway was important in nasopharyngeal carcinogenesis, and that the expression of CD99 in lymphoid stroma might regulate immune response to NPC [80].

LMP-1 played an important role in enhancing NPC cell response to arsenic trioxide (As_2_O_3_). The elongation of telomere length induced by LMP-1 might contribute to the mechanisms of As_2_O_3 _sensitivity [81]. Preclinical studies demonstrated that As_2_O_3 _could inhibit LMP-1 expression, dictate apoptosis and alterations of cell cycle distribution and growth retardation. LMP-1-positive NPC cells were more sensitive to As_2_O_3 _treatment than LMP-1-negative NPC cells [82]. Further study found that As_2_O_3 _could reduce metastatic potential of NPC cells, involving inhibition of MMP-9 expression. LMP-1 were reduced in this process and seemed to enhance anti-metastatic activity of As_2_O_3 _[83].

It was suggested that nasopharyngeal swab could be effective method for gene detection. As a parameter in diagnosis of NPC, *LMP-1 *might be superior to VCA-IgA. Thirty-bp deletion of *LMP-1 *was widespread in NPC patients [84]. The nasopharyngeal swab coupled with PCR based EBV *LMP-1 *and *EBNA *detection could serve as a good supplement to pathological diagnosis of NPC [85].

EBV-encoded *LMP-1 *was vulnerable to RNAi and selective inhibition of *LMP-1 *had anti-proliferation effect on NPC cell. RNAi could be a powerful tool in further investigations of *LMP-1 *and a novel therapeutic strategy for EBV-associated NPC patients [86]. A recombinant adeno-associated virus type 2 vector was used to deliver shRNA targeting EBV *LMP-1 *into the EBV-positive human NPC C666-1 cells. Results demonstrated that long-term suppression of EBV-encoded *LMP-1 in vivo *is an effective means for preventing NPC metastasis [87].

## Emerging perspectives

Contemporary NPC research has distincted itself from the traditional one with the unprecedented large amount of data and tremendous diagnostic and therapeutic innovations. Data are currently generated in high-throughput fashions with the integration and application of systems biology. Genomics, proteomics, metabolomics and bioinformatics each plays a more and more important role for molecular biomarker discovery [88]. We now have a better understanding of the disease, including its diagnosis, monitoring, treatment and prognostication. In the era of molecular targeted therapy, specific treatment to the potential target using technologies such as immunotherapy and RNAi becomes formulating from bench to bedside application and thus makes molecular biomarker discovery more meaningful for NPC management.

**Table 1:** Biomarkers identified by proteomics technologies in nasopharyngeal carcinoma

**Technology**	**Primary use**	**Biomarker**
Two-dimensional electrophoresis and matrix-assisted laser desorption/ionization time-of-flight mass spectrometry	Diagnosis	Fibronectin, Mac-2 binding protein, Plasminogen activator inhibitor 1
	Signaling target	Annexin A2, Heat shock protein 27, Stathmin, Annexin I, Basic transcription factor 3, Porin
	Treatment response monitoring	Ceruloplasmin
Surface-enhanced laser desorption/ionization time-of-flight mass spectrometry and tandem mass spectrometry	Diagnosis	Inter-α-trypsin inhibitor precursor
	Treatment response monitoring	Platelet factor-4
	Prognosis	Serum amyloid A
